# Performance of Regression-Based Norms for Cognitive Functioning of Persons With Multiple Sclerosis in an Independent Sample

**DOI:** 10.3389/fneur.2020.621010

**Published:** 2021-01-14

**Authors:** Ruth Ann Marrie, Christiane E. Whitehouse, Ronak Patel, Chase R. Figley, Jennifer Kornelsen, James M. Bolton, Lesley A. Graff, Erin L. Mazerolle, James J. Marriott, Charles N. Bernstein, John D. Fisk

**Affiliations:** ^1^Department of Internal Medicine, Rady Faculty of Health Sciences, Max Rady College of Medicine, University of Manitoba, Winnipeg, MB, Canada; ^2^Department of Community Health Sciences, Rady Faculty of Health Sciences, Max Rady College of Medicine, University of Manitoba, Winnipeg, MB, Canada; ^3^Department of Psychology & Neuroscience, Dalhousie University, Halifax, NS, Canada; ^4^Department of Clinical Health Psychology, Rady Faculty of Health Sciences, Max Rady College of Medicine, University of Manitoba, Winnipeg, MB, Canada; ^5^Department of Radiology, Rady Faculty of Health Sciences, Max Rady College of Medicine, University of Manitoba, Winnipeg, MB, Canada; ^6^Division of Diagnostic Imaging, Winnipeg Health Sciences Centre, Winnipeg, MB, Canada; ^7^Neuroscience Research Program, Winnipeg Health Sciences Centre, Kleysen Institute for Advanced Medicine, Winnipeg, MB, Canada; ^8^Department of Psychiatry, Rady Faculty of Health Sciences, Max Rady College of Medicine, University of Manitoba, Winnipeg, MB, Canada; ^9^Department of Psychology, St. Francis Xavier University, Antigonish, NS, Canada; ^10^Departments of Psychiatry, and Medicine, Dalhousie University, Halifax, NS, Canada

**Keywords:** multiple sclerosis, cognition, regression-based norms, reliability, BICAMS

## Abstract

**Background:** Cognitive impairment is common in multiple sclerosis (MS). Interpretation of neuropsychological tests requires the use of normative data. Traditionally, normative data have been reported for discrete categories such as age. More recently continuous norms have been developed using multivariable regression equations that account for multiple demographic factors. Regression-based norms have been developed for use in the Canadian population for tests included in the MACFIMS and BICAMS test batteries. Establishing the generalizability of these norms is essential for application in clinical and research settings.

**Objectives:** We aimed to (i) test the performance of previously published Canadian regression-based norms in an independently collected sample of Canadian healthy controls; (ii) compare the ability of Canadian and non-Canadian regression-based norms to discriminate between healthy controls and persons with MS; and (iii) develop regression-based norms for several cognitive tests drawn from batteries commonly used in MS that incorporated race/ethnicity in addition to age, education, and sex.

**Methods:** We included 93 adults with MS and 96 healthy adults in this study, with a replication sample of 104 (MS) and 39 (healthy adults). Participants reported their sociodemographic characteristics, and each was administered the oral Symbol Digit Modalities Test (SDMT), the California Verbal Learning Test (CVLT-II), and the Brief Visuospatial Memory Test-Revised (BVMT-R). From the healthy control data, we developed regression-based norms incorporating race, age, education and sex. We then applied existing discrete norms and regression-based norms for the cognitive tests to the healthy controls, and generated z-scores which were compared using Spearman rank and concordance coefficients. We also used receiver operating characteristic (ROC) curves to compare the ability of each set of norms to discriminate between participants with and without MS. Within the MS samples we compared the ability of each set of norms to discriminate between differing levels of disability and employment status using relative efficiency.

**Results:** When we applied the published regression norms to our healthy sample, impairment classification rates often differed substantially from expectations (7%), even when the norms were derived from a Canadian (Ontario) population. Most, but not all of the Spearman correlations between z-scores based on different existing published norms for the same cognitive test exceeded 0.90. However, concordance coefficients were often lower. All of the norms for the SDMT reliably discriminated between the MS and healthy control groups. In contrast, none of the norms for the CVLT-II or BVMT-R discriminated between the MS and healthy control groups. Within the MS population, the norms varied in their ability to discriminate between disability levels or employment status; locally developed norms for the SDMT and CVLT-II had the highest relative efficiency.

**Conclusion:** Our findings emphasize the value of local norms when interpreting the results of cognitive tests and demonstrate the need to consider and assess the performance of regression-based norms developed in other populations when applying them to local populations, even when they are from the same country. Our findings also strongly suggest that the development of regression-based norms should involve larger, more diverse samples to ensure broad generalizability.

## Introduction

Over 40% of persons with multiple sclerosis (MS) are thought to experience cognitive impairment which adversely affects social participation, independence, and employment (1, 2). Cognitive impairment at diagnosis has been found to be associated with disability progression over time (3). Neuropsychological assessments objectively evaluate cognitive function, and are increasingly important in the care of persons with MS patients, as new rehabilitative strategies and pharmacologic therapies for cognitive impairments continue to emerge. Given that access to comprehensive neuropsychological assessments is often limited, several abbreviated test batteries have been recommended for use in persons with MS, including the Brief International Cognitive Assessment for Multiple Sclerosis (BICAMS) (4). Brief Repeatable Battery of Neuropsychological Tests (BRB-N) (5), and the Minimal Assessment of Cognitive Function in MS (MACFIMS) (5, 6). Interpretation of test results for both research and for clinical practice requires the use of normative data, although most available published normative data for these tests were developed in American populations. Application of American norms to Canadian populations is not recommended due to differences in performance between Canadian and American adults on measures of intellectual ability (7). Moreover, published norms were often established in samples that no longer reflect contemporary demographics; for example the proportion of individuals with higher levels of education was lower than in the present day population. Notably, Intelligence Quotient scores have risen over time (8), and use of outdated norms may lead to misclassification of cognitive status by underestimating the normal range of performance (9). In consideration of these issues, recommendations for international validation of the BICAMS were made to encourage its adoption (10).

Traditionally, normative data have been reported for discrete categories, such as age and/or education. More recently, continuous norms have been developed using multivariable regression equations that account for multiple demographic factors simultaneously. Regression-based norms for use in the Canadian population were recently developed for tests included in the MACFIMS battery (11), including the subset of tests included in BICAMS. Because these norms were derived from control populations recruited for other purposes, the number of participants available was fewer than the recommended 100 participants for some tests. In addition, while developed for use in Canada, the controls were drawn from only one region of Canada (i.e., province of Ontario), and the performance of these norms in an independently collected sample of healthy Canadian persons has not yet been assessed. Establishing the generalizability of norms is essential to determine if they may be appropriately applied in clinical and research settings more broadly than those from which the normative samples were drawn.

We sought to (i) test the performance of the previously published Canadian (Ontario) regression-based norms in independently collected samples of healthy controls from other Canadian regions; (ii) develop local regression-based norms for the tests included in the BICAMS; and (iii) examine differences in impairment classification rates in local healthy controls when applying BICAMS regression-based norms from different populations; and (iv) examine the ability of Canadian and non-Canadian norms to discriminate between local healthy and MS samples.

## Methods

We conducted the primary analysis using MS and healthy control samples from Manitoba, Canada. Manitoba is a central Canadian province with a population of ~1.4 million people. We replicated our analyses in MS and healthy control samples from the eastern Canadian province of Nova Scotia (population ~1.0 million), which are described further in the replication section.

### Setting and Participants

In Manitoba, we enrolled a subgroup of persons with MS participating in a longitudinal study of immune-mediated inflammatory diseases (the “IMID” study) as previously described (12). Participants were recruited from the single specialized care center for persons with MS in the province. This subgroup of 111 participants attended an IMID study visit between September 2016 and July 2017 which included cognitive testing (13). MS participants were aged ≥18 years, with adequate knowledge of the English language to provide informed consent.

We enrolled healthy controls from September 2018 to September 2019. Inclusion criteria for study participation included aged ≥18 years, with adequate knowledge of the English language to provide informed consent. Exclusion criteria included any chronic medical condition, known cognitive impairment, any positive response to the Structured Clinical Interview for DSM-IV (SCID-IV) screening questions for depressive or anxiety disorders, any head injury associated with loss of consciousness or amnesia, or chronic medication use with the exceptions of contraceptives, hormone replacement therapy, transient antibiotic use, or multivitamins (14). Hypertension, as identified during the study visit (see below), was also an exclusion criterion even if not reported as a diagnosed condition by the participant. We recruited participants using multiple methods including posters placed in hospital, university, and community settings throughout Winnipeg; mail-outs of a study poster to homes in Winnipeg; and word of mouth. Sample size requirements for the development of regression-based norms are 2.5 to 5.5-fold smaller than for the development of discrete norms, while retaining similar or better precision (15), and samples of 100–500 persons are sufficient. Thus, our target sample size was 100.

### Participant Characteristics

All participants, including those with MS and healthy participants, underwent standardized assessments and completed questionnaires (12). Participants reported their sociodemographic characteristics including sex, date of birth, ethnicity, years of education, and annual household income as described in detail previously (12). Participants also reported their smoking status; we classified participants who had smoked at least 100 cigarettes as ever smokers (16). We determined body mass index (BMI, kg/m^2^) based on height and weight measured at the study visit. Only participants with MS underwent a neurological examination for calculation of the Expanded Disability Status Scale (EDSS) score by an EDSS-certified neurologist.

### Neuropsychological Measures

We were primarily interested in the development of local regression-based norms to support an ongoing study examining the influence of vascular and psychiatric comorbidity on cognition in MS (13). The neuropsychological tests conducted examined cognitive domains most often affected in MS, and the comorbidities of interest (17, 18) and included tests of information processing speed, verbal learning and memory, and visual learning and memory. From these tests we examined the test scores comprising the BICAMS, i.e., the oral Symbol Digit Modalities Test (SDMT) (19), the California Verbal Learning Test (CVLT-II; Trial 1–5 total recall score) (20), and the Brief Visuospatial Memory Test-Revised (BVMT-R; summed recall score for all three learning trials) (21). Each participant also completed the Wechsler Test of Adult Reading (WTAR) as an estimate of premorbid IQ.

### Analyses

First, we summarized participant characteristics using descriptive characteristics including mean, standard deviation (SD), frequency and percent (%).

Second, to develop regression-based norms in our healthy control group we adapted the approach previously described by Berrigan et al. (22) Specifically, we converted raw scores to scaled scores with a mean of 10 and standard deviation (SD) of 3 based on the cumulative frequency distribution in our control group. Then, we developed a separate regression model for each test or subtest of interest, where the scaled test score was the dependent variable. To account for the bounded distribution of the scaled scores and ensure that predicted values did not fall outside the range of possible values, we used truncated rather than linear regression models. The independent variables were sex (coded as 1 = male, 2 = female), years of education (continuous), age (continuous), age-squared (continuous), and race/ethnicity (coded as 1 = white, 0 = non-white). We included an age-squared term to account for potential non-linear relationships (22). We included race/ethnicity given that cognitive tests may assess individuals of different racial backgrounds differently (23, 24). We did not include estimated pre-morbid IQ as this variable was not included in the development of regression-based norms in MS. For consistency with published Canadian norms, we also report norms without this predictor, and in individuals aged 65 years and under. For each regression model we report the constant and non-standardized coefficients that generate the normative formulae. Model fit was assessed using a pseudo-*R*^2^ calculated as the squared correlation of the observed and predicted values of the dependent variable (25). We assessed assumptions of homoscedasticity using the White test and residual plots, and assessed assumptions of normality using quantile-quantile plots.

Third, we applied previously published regression-based Canadian norms for the tests where available (11, 22). Two sets of norms were available for the SDMT; we tested both the norms developed using only Ontario participants (11) and the norms developed using participants from Ontario and Nova Scotia (hereinafter Ontario/Nova Scotia) (22). Because these norms were developed in persons aged 18 to 65 years (Supplementary Table e1), and accordingly may not perform adequately in older participants, we excluded study participants over age 65 years when examining their performance. Z-scores of ≤-1.5 were classified as impaired. We expected that if the norms performed well, based on a normal distribution ~7% of our healthy control sample would be classified as impaired on each test.

Fourth, we compared the Canadian regression based norms with non-Canadian regression based norms after applying the norms to generate z-scores. Other norms examined included regression-based norms developed in two other English-speaking populations [Buffalo, New York, United States (hereafter “New York”); Dublin, Ireland (hereafter “Ireland”)] (26), the discrete norms available from the published test manuals for each test, and the recently published discrete norms for the SDMT by Strober et al. which were intended to update the previous discrete norms (27). We did not examine regression-based norms for BICAMS developed in non-English-speaking populations (28). The characteristics of the samples used to develop these norms are shown in Supplementary Table e1. For these comparisons, we examined the Spearman correlations between the z-scores. We considered correlations of ≤0.39 as low, 0.40–0.59 as moderate, 0.60–0.79 as strong, and ≥0.80 as very strong (29). Because Spearman correlations can establish whether the rank order of participant z-scores are the same, but not whether the same z-score values are assigned, we also examined the concordance coefficients (30). In order to assess the ability of the various norms to differentially discriminate between persons with MS and healthy individuals we compared the area under the receiver operating characteristic (ROC) curve between the various norms, using binary logistic regression, where the dependent variable was MS vs. healthy participant classification.

Given prior reports of an increased frequency of cognitive impairment in persons with MS at greater levels of disability, we examined the ability of each set of norms to discriminate between differing levels of neurologic disability amongst the MS sample (31). We categorized MS participants according to their EDSS scores into mild (0–2.5), moderate (3.0–4.0), and severe (≥4.5) disability groups. We also examined the ability of the norms to discriminate between employed and unemployed persons with MS, where employment status was determined based on the Work Productivity and Impairment Scale (32). Discriminating ability was examined using relative efficiency (RE), where the RE of each set of norms was calculated as the ratio of between group (3 EDSS levels; or 2 employment categories) ANOVA F-statistics. The largest F-statistic represents the greatest discriminative ability.

### Replication

Data from an independent sample of MS participants and healthy controls, collected in Nova Scotia, Canada, were used to repeat the analyses comparing Canadian and non-Canadian regression-based norms, including correlations between the norms and their ability to discriminate between healthy and MS samples. These participants were enrolled in an ongoing longitudinal study of attention network functioning in MS and were recruited from the single specialized MS care center in that province. Unlike the Manitoba sample, these MS participants were selected to have an EDSS <4.5, with an age range from 20 to 60 years old. Exclusion criteria included insufficient visual acuity or impaired dexterity that would impede performance on cognitive tasks) or comorbid conditions that were likely to have a significant impact on their cognition (e.g., neurologic disorders other than MS, diagnosed learning disability, previous head injury with loss of consciousness, and sub-optimally managed psychiatric disorder as determined by clinic staff). As the independent Nova Scotia sample was selected to have no more than moderate levels of neurologic disability, only one participant fell within the “severe” EDSS category of >4.5 used in the previous analyses. Therefore, these participants were instead divided into only two categories: mild (0–2.5) and moderate (3.0–4.5). The data of 104 MS participants, tested between August 2016 and July 2018, were used in the current study replication. Healthy control participants (*n* = 39) recruited over this time period met the same exclusion criteria as the MS group but had no history or family history of MS and no history of psychiatric disorder; they were matched to the MS group based on age, years of education, and sex. Although all necessary cognitive measures were available in this dataset, several demographic variables were not collected: Ethnicity, annual household income, smoking status, and body mass index.

Statistical analyses were conducted using SAS V9.4 (SAS Institute Inc., Cary, NC) and SPSS Version 25 (IBM Corp., Armonk, NY).

## Results

Throughout, we present the findings in Manitoba followed by the findings in the Nova Scotia replication sample. Of the 103 healthy participants from Manitoba, 96 were under age 65 years, and of 111 participants with MS, 93 were under age 65 years. The healthy participants were younger on average, but the age range of the healthy participants (18.2–64.4) was similar to that of the participants with MS (20.8–63.8) years. Most participants in each group were women, although the proportion who were women was higher in the MS group (Table 1). The average number of years of education was consistent with at least some post-secondary education in both groups although the healthy control group averaged 2.4 more years of education than the MS group. Race/ethnicity did not differ between the two groups, nor did estimated household income.

**Table 1:** Characteristics of participants.

**Characteristic**					
**Manitoba**	**Healthy (all)**	**Healthy ≤65 years**	**MS**	**Std Diff^a^**	***P*-value^a^**
*N*	103	96	93		
Age (year), mean (SD)	38.7 (16.3)	36.1 (13.6)	45.6 (9.6)	0.81	<0.0001
Women, *n* (%)	68 (66.0)	64 (66.7)	77 (82.8)	0.16	0.011
White, *n* (%)	85 (82.5)	79 (82.3)	74 (80.4)	0.02	0.74
Years of education, mean (SD)	16.7 (3.0)	16.6 (3.0)	14.2 (2.6)	0.85	<0.0001
Annual income, *n* (%)					0.48
< $50,000	33 (32.0)	32 (33.3)	26 (28.0)	0.053	
≥$50,000	60 (58.3)	55 (57.3)	61 (65.6)	0.083	
I do not wish to answer	10 (9.7)	9 (9.4)	6 (6.4)	0.03	
Employed^b^, *n* (%)	82 (79.6)	81 (81.4)	54 (58.7)	0.23	<0.0001
Ever Smoker, *n* (%)	13 (12.6)	12 (18.2)	54 (58.1)	0.40	<0.0001
BMI (kg/m^2^), mean (SD)	25.5 (4.7)	25.4 (4.7)	29.0 (6.6)	0.63	<0.0001
FSIQ, mean (SD)	110 (7.8)	109.9 (7.7)	106.3 (8.2)	0.45	0.0022
**Nova Scotia**	**Healthy**		**MS**	**Std Diff^c^**	***P*****-value^c^**
*N*	39		104		
Age (year), mean (SD)^d^	49.4 (9.7)		47.0 (8.6)	0.27	0.19
Women, *n* (%)	35 (89.7)		91 (87.5)	0.07	0.71
Years of education, mean (SD)	15.1 (1.5)		14.6 (1.8)	0.29	0.11

a *For comparison of healthy (n = 96) and MS (n = 93) participants aged 65 years and under in Manitoba*;

b *missing for one person with MS*;

c *For comparison of healthy (n = 39) and MS (n = 104) participants in Nova Scotia*;

d *all healthy participants < age 60 years*.

In the replication sample, most participants were also women, and the average number of years of education was consistent with at least some post-secondary education (Table 1).

### Impairment Classification Rates

Table 2 shows raw score to scaled score conversions used to develop the regression-based norms in healthy controls aged 65 years and younger in Manitoba. Table 3 shows the regression-based formulae with and without race as a covariate. The degree of variance in the cognitive tests explained by demographic factors varied slightly between tests.

**Table 2:** Raw score to scaled score conversions.

**Scaled Score**	**SDMT**	**CVLT-II verbal learning**	**BVMT-R total recall**
2	<40	<30	<10
3	40–43	30–31	10–11
4	44–46	32–38	12
5	47–50	39–40	13–14
6	51–54	41–43	15–19
7	55–57	45–47	20–22
8	58–59	48–49	23–24
9	60–62	50–55	25–26
10	63–65	56–57	27–28
11	66–69	58–61	29–30
12	70–73	62–63	31–32
13	74–78	64	33
14	79–80	65–68	34
15	81	69	35
16	82–83	70	36
17	≥84	>70	
18			

**Table 3:** Regression-based norms with and without incorporating race as a demographic predictor derived from healthy controls aged ≤65 years^a^.

**Test**	**Constant**	**Std Err**	**Sex**	**Age**	**Age^**2**^**	**Educ**	**Race**	**Pseudo-*R^**2**^***
SDMT	8.16	2.62	0.74, *p* = 0.20	−0.088, *p* = 0.0005	0.002, *p* = 0.19	0.005, *p* = 0.96		0.15
SDMT	6.76	2.52	0.98, *p* = 0.08	−0.080, *p* = 0.0012	0.002, *p* = 0.21	−0.024, *p* = 0.80	1.84, *p* = 0.0082	0.21
CVLT-II, verbal learning	3.81	2.71	1.96, *p* = 0.0009	−0.024, *p* = 0.36	0.002, *P* = 0.39	0.15, *P* = 0.13		0.13
CVLT-II, verbal learning	2.99	2.68	2.11, *p* = 0.0004	−0.019, *p* = 0.46	0.002, *p* = 0.42	0.14, *p* = 0.18	1.09, *p* = 0.14	0.15
BVMT-R, total recall	8.45	2.53	1.02, *p* = 0.066	−0.084, *p* = 0.0007	0.001, *p* = 0.45	−0.028, *p* = 0.77		0.18
BVMT-R, total recall	7.51	2.49	1.18, *p* = 0.032	−0.078, *p* = 0.0014	0.001, *p* = 0.48	−0.048, *p* = 0.61	1.24, *p* = 0.069	0.21

a *Truncated regression; Sex 1 = male, 2 = female; age in years and centered at 36.12; education in years; SDMT, Symbol Digit Modalities Test; CVLT-II, California Verbal Learning Test; BVMTR, Brief Visual Memory Test-Revised*.

**Figure 1:**
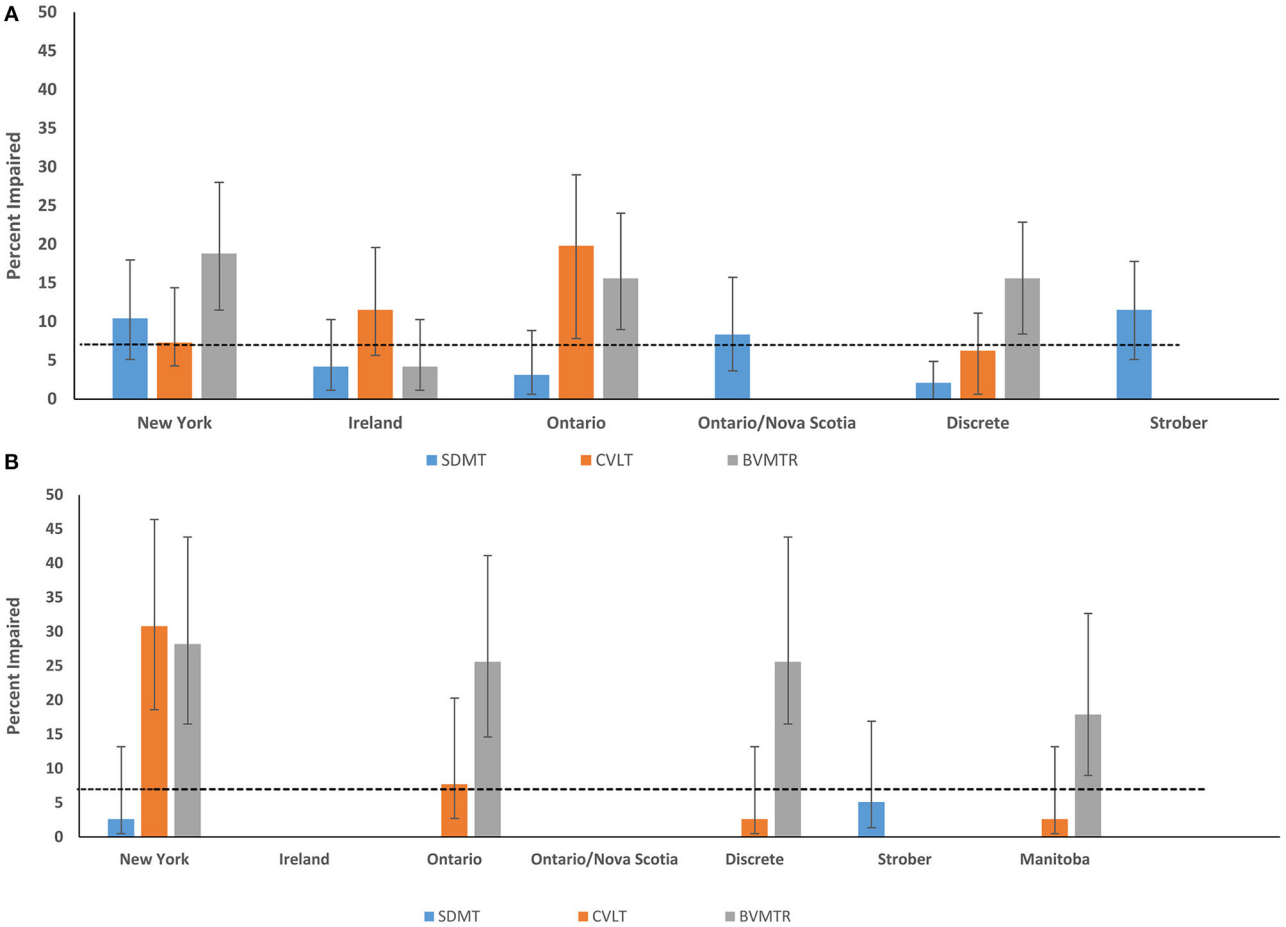
Impairment rates in Healthy Control participants according to regression-based and discrete norms from English-speaking populations **(A)** Manitoba **(B)** Nova Scotia. SDMT, Symbol Digit Modalities Test; CVLT-II, California Verbal Learning Test; BVMTR, Brief Visual Memory Test-Revised.

When we applied the published regression norms to the healthy Manitoba sample, the impairment classification rates often differed substantially from the expected rate of 7%, even when the norms were derived from another Canadian (Ontario) population. The exceptions for the SDMT were the regression-based norms from Ontario/Nova Scotia and New York; and for the CVLT were the regression-based norms from New York, and the discrete norms (Figure 1A).

When the published regression norms and locally developed Manitoba norms were applied to the independent Nova Scotia healthy sample, impairment classification rates were lower and more often within the expected range based on a normal population distribution (i.e., 7%) (Figure 1B). However, there were notable outliers: 30.8% and 28.2% of controls in the replication sample of healthy controls were impaired on the CVLT-II and BVMT-R, respectively, using the New York norms; 25.6% were impaired on BVMT-R using the Ontario norms; and 25.6% impaired on the BVMT-R using the discrete norms.

### Correlations and Concordance Between Norms

In the Manitoba sample, most, but not all of the Spearman correlations between z-scores based on existing published norms for the same cognitive test exceeded 0.90 (Table 4). However, concordance coefficients were often lower, ranging from 0.45 to 0.96 (Table 4). The discrepancies between norms appeared to be greatest between the norms from Ireland as compared to all other norms. This pattern of high correlation coefficients, with the greatest discrepancies between the norms from Ireland and other norms, was replicated in the independent Nova Scotia sample (Supplementary Table e2). In addition, correlations between the locally developed Manitoba norms and all other norms showed the same pattern.

**Table 4:** Spearman two-tailed correlation coefficients and concordance coefficients for the association between different norms in Manitoba.

	**Healthy controls (*****n*** **=** **96)**		**Multiple sclerosis (*****n*** **=** **93)**	
	**Correlation coefficient (95% CI)**	**Concordance coefficient (95% CI)**	**Correlation coefficient (95% CI)**	**Concordance coefficient (95% CI)**
**SDMT**				
SDMT_NY_-SDMT_IRE_	0.88 (0.83, 0.92)	0.66 (0.57, 0.74)	0.94 (0.91, 0.96)	0.70 (0.62, 0.77)
SDMT_NY_-SDMT_ONT_	0.96 (0.94, 0.98)	0.86 (0.90, 0.93)	0.96 (0.94, 0.97)	0.91 (0.87, 0.94)
SDMT_NY_-SDMT_ONT/NS_	0.98 (0.97, 0.99)	0.96 (0.95, 0.98)	0.97 (0.96, 0.98)	0.93 (0.90, 0.95)
SDMT_IRE_-SDMT_ONT_	0.86 (0.80, 0.91)	0.73 (0.65, 0.80)	0.93 (0.90, 0.95)	0.81 (0.74, 0.86)
SDMT_IRE_-SDMT _ONT/NS_	0.89 (0.84, 0.93)	0.65 (0.74, 0.80)	0.91 (0.87, 0.94)	0.76 (0.68, 0.82)
SDMT_ONT_-SDMT _ONT/NS_	0.97 (0.96, 0.98)	0.95 (0.93, 0.97)	0.97 (0.95, 0.98)	0.96 (0.94, 0.97)
SDMT_NY_-SDMT_DISCRETE_	0.91 (0.87, 0.94)	0.83 (0.77, 0.88)	0.93 (0.90, 0.95)	0.83 (0.77, 0.88)
SDMT_NY_-SDMT_STROBER_	0.92 (0.89, 0.95)	0.86 (0.81, 0.90)	0.92 (0.88, 0.95)	0.90 (0.86, 0.94)
SDMT_IRE_-SDMT_DISCRETE_	0.87 (0.81, 091)	0.72 (0.63, 0.78)	0.92 (0.88, 0.95)	0.82 (0.76, 0.87)
SDMT_IRE_-SDMT_STROBER_	0.76 (0.65, 0.83)	0.69 (0.83, 0.78)	0.87 (0.81, 0.92)	0.74 (0.65, 0.82)
SDMT_ONT_-SDMT_DISCRETE_	0.96 (0.94, 0.97)	0.96 (0.93, 0.97)	0.97 (0.96, 0.98)	0.96 (0.94, 0.97)
SDMT_ONT_-SDMT_STROBER_	0.92 (0.88, 0.95)	0.89 (0.84, 0.92)	0.95 (0.91, 0.96)	0.92 (0.88, 0.95)
SDMT_NS_-SDMT_DISCRETE_	0.93 (0.90, 0.96)	0.90 (0.86, 0.93)	0.94 (0.90, 0.96)	0.92 (0.88, 0.94)
SDMT_DISCRETE_-SDMT_STROBER_	0.93 (0.89, 0.95)	0.91 (0.87, 0.94)	0.92 (0.87, 0.95)	0.90 (0.86, 0.93)
**CVLT**				
CVLT_NY_-CVLT_IRE_	0.74 (0.64, 0.82)	0.68 (0.57, 0.76)	0.83 (0.76, 0.89)	0.73 (0.64, 0.80)
CVLT_NY_-CVLT_ONT_	0.87 (0.82, 0.91)	0.79 (0.71, 0.85)	0.94 (0.91, 0.96)	0.92 (0.88, 0.94)
CVLT_IRE_-CVLT_ONT_	0.94 (0.91, 0.96)	0.80 (0.74, 0.85)	0.94 (0.91, 0.96)	0.80 (0.74, 0.85)
CVLT_NY_-CVLT_DISCRETE_	0.93 (0.89, 0.95)	0.83 (0.77, 0.88)	0.96 (0.94, 0.97)	0.90 (0.86, 0.93)
CVLT_IRE_-CVLT_DISCRETE_	0.86 (0.79, 0.72)	0.80 (0.72, 0.85)	0.89 (0.82, 0.92)	0.84 (0.78, 0.88)
CVLT_ONT_-CVLT_DISCRETE_	0.90 (0.85, 0.93)	0.65 (0.57, 0.73)	0.94 (0.91, 0.96)	0.82 (0.76, 0.87)
**BVMTR**				
BVMTR_NY_-BVMTR_IRE_	0.85 (0.79, 0.90)	0.45 (0.36, 0.53)	0.87 (0.81, 0.91)	0.36 (0.28, 0.43)
BVMTR_NY_-BVMTR_ONT_	0.90 (0.85, 0.93)	0.85 (0.80, 0.89)	0.92 (0.88, 0.95)	0.85 (0.79, 0.89)
BVMTR_IRE_-BVMTR_ONT_	0.97 (0.96, 0.98)	0.51 (0.43, 0.58)	0.96 (0.95, 0.98)	0.49 (0.41, 0.56)
BVMTR_NY_-BVMTR_DISCRETE_	0.85 (0.78. 0.90)	0.66 (0.75, 0.81)	0.88 (0.82, 0.92)	0.75 (0.67, 0.82)
BVMTR_IRE_-BVMTR_DISCRETE_	0.97 (0.95, 0.98)	0.68 (0.60, 0.74)	0.96 (0.93, 0.97)	0.59 (0.51, 0.66)
BVMTR_ONT_-BVMTR_DISCRETE_	0.98 (0.97, 0.99)	0.91 (0.88, 0.94)	0.99 (0.98, 0.99)	0.96 (0.94, 0.97)

*SDMT, Symbol Digit Modalities Test; CVLT-II, California Verbal Learning Test; BVMTR, Brief Visual Memory Test-Revised; ONT, Ontario; NY, New York; NS, Nova Scotia; IRE, Ireland*.

### Ability of BICAMS Norms to Discriminate Between MS and Healthy Control Groups

**Figure 2:**
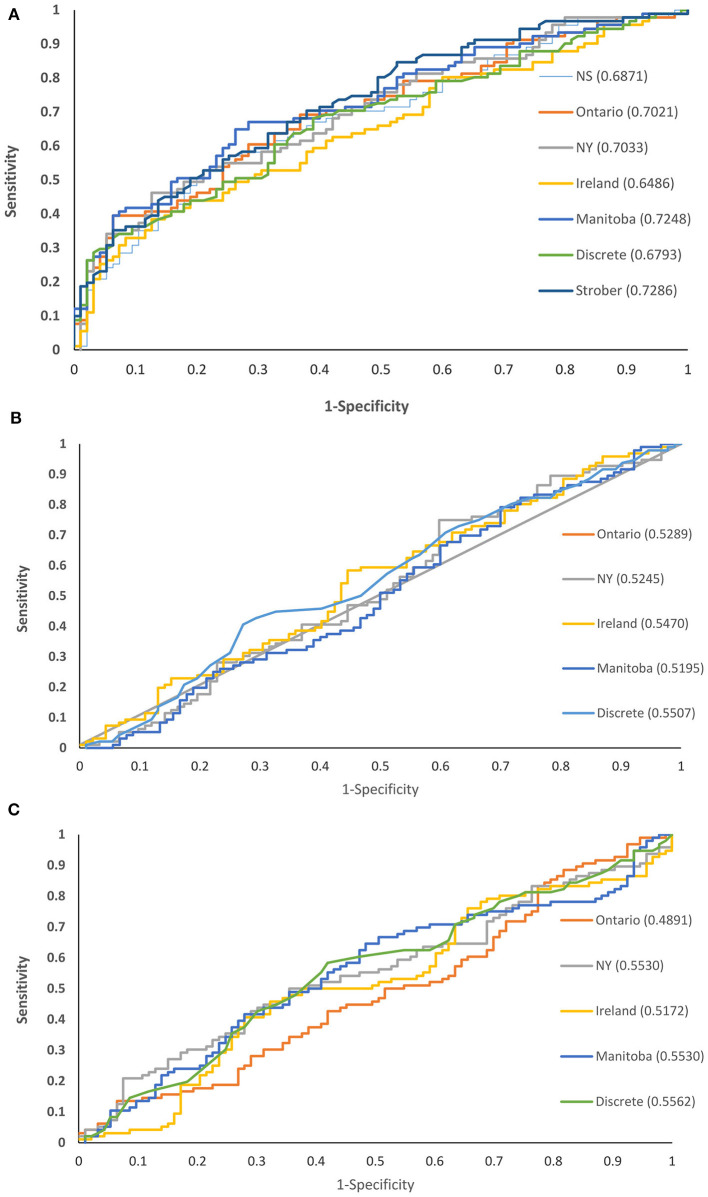
Receiver operating characteristic curves for cognitive test norms comparing persons with and without multiple sclerosis in Manitoba: **(A)** SDMT **(B)** CVLT-II **(C)** BVMT-R.

All of the norms for the SDMT discriminated between the MS and healthy control groups, based on ROC analyses, but they differed in their ability to do so (Figure 2A). The area under the ROC curve (AUC) was highest for the Strober et al. discrete norms and the locally developed Manitoba norms (without race for comparability), and the AUC was lowest for the Irish norms. As compared to the Manitoba norms (AUC 0.72; 95% CI: 0.65–0.80), the Strober (AUC 0.73; 95% CI: 0.66–0.80, *p* = 0.81) and New York norms (AUC 0.70; 95% CI: 0.63–0.78, *p* = 0.18) did not differ. As compared to the Manitoba norms, the Ontario (AUC 0.70; 95% CI: 0.63–0.78, *p* = 0.01), Ontario/Nova Scotia (AUC 0.69; 95% CI: 0.61–0.76, *p* = 0.0038), Irish (AUC 0.65; 95% CI: 0.57–0.73, *p* = 0.0002) and discrete norms from the SDMT manual (AUC 0.68; 95% CI: 0.60–0.76, *p* < 0.0001) did not discriminate as well.

None of the norms for the CVLT-II verbal learning discriminated between the MS and healthy control groups (Figure 2B). The discriminating ability of the Manitoba norms (AUC 0.50; 95% CI: 0.42–0.59) did not differ from that of the Ontario (AUC 0.53; 95% CI: 0.45–0.62, *p* = 0.68), New York (AUC 0.52; 95% CI: 0.44–0.61, *p* = 0.32), Irish (AUC 0.55; 95% CI: 0.63–0.63, *p* = 0.52) or discrete (AUC 0.55; 95% CI: 0.47–0.63, *p* = 0.057) norms.

None of the norms for the BVMT-R total recall discriminated between the MS and healthy control groups (Figure 2C). The discriminating ability of the Manitoba norms (AUC 0.55; 95% CI: 0.47–0.64) did not differ from that of the Ontario (AUC 0.49; 95% CI: 0.41–0.57, *p* = 0.44), New York (AUC 0.55; 95% CI: 0.47–0.64, *p* = 1.0), Irish (AUC 0.52; 95% CI 0.43–0.60, *p* = 0.083) or discrete (AUC 0.56; 95% CI: 0.47–0.64, *p* = 0.78) norms.

Similarly, based on ROC analyses of the independent Nova Scotia sample, all norms for the SDMT discriminated between the MS and healthy control groups, while none of the norms for the BVMT-R total recall discriminated between groups (Supplementary Figure e1). However, unlike the Manitoba sample, all norms for the CVLT-II verbal learning did discriminate between MS and healthy control groups.

### Ability of Different Norms to Discriminate Between MS Participants With Differing Levels of Disability or Employment Status

We next examined whether application of the various norms influenced the extent to which the tests discriminated between differing levels of disability based on the EDSS, amongst individuals within the Manitoba MS cohort. For the SDMT, the Manitoba norms were best able to discriminate between disability groups (Table 5). The relative efficiency (RE) for the Ontario and Strober norms exceeded 0.92 compared to the Manitoba norms but the remaining norms had substantially lower RE of 0.52–0.54. For the CVLT-II verbal learning, the Manitoba norms were again best able to discriminate between disability groups. The New York norms had a similar discriminating ability with a RE of 0.97. The remaining norms had lower RE of 0.36–0.69. For the BVMT-R total recall, the discrete norms had the best discriminating ability, while the New York norms had the lowest RE. Considering only the Manitoba norms, the BVMT-R best discriminated between differing disability levels, followed by the SDMT and CVLT-II. This same pattern was seen for the Ontario, Ireland and discrete norms from the manual, but not for the New York norms where the BVMT-R had the poorest discriminating ability.

**Table 5:** Ability of various norms to discriminate differing levels of disability and employment status among participants with MS.

**Manitoba sample (*****N*** **=** **93)**							
		**Disability**			**Employment**		
**Test**	**Norm**	***F*-test**	***P*-value**	**Relative efficiency**	***F*-test**	***P*-value**	**Relative efficiency**
SDMT	Manitoba	10.2	0.0001	1	5.19	**0.025**	1
	Ontario	9.38	0.0002	0.92	4.42	**0.038**	0.85
	Ontario/Nova Scotia	5.46	0.0058	0.54	1.90	0.17	0.37
	Strober	8.07	0.0006	0.79	2.98	0.088	0.57
	Discrete	7.82	0.0007	0.54	4.19	**0.044**	0.81
	New York	5.3	0.0067	0.52	2.91	0.092	0.56
	Ireland	5.21	0.0072	0.51	4.64	**0.034**	0.89
CVLT-II	Manitoba	8.45	0.0004	1	3.72	0.057	0.95
	Ontario	5.87	0.004	0.69	3.90	0.051	1
	Discrete	5.49	0.0056	0.65	3.13	0.080	0.80
	New York	8.16	0.0006	0.97	2.69	0.10	0.69
	Ireland	3.08	0.051	0.36	3.05	0.084	0.78
BVMT-R	Manitoba	12.99	<0.0001	0.79	6.92	**0.01**	0.60
	Ontario	14.12	0.0001	0.86	8.16	**0.0053**	0.71
	Discrete	16.45	<0.00001	1	11.54	**0.001**	1
	New York	6.38	0.0026	0.39	1.92	0.17	0.17
	Ireland	13	<0.0001	0.86	9.92	**0.0022**	0.86
	**Nova Scotia Sample (N** **=** **104)** **Disability^a^**						
SDMT	Manitoba	15.032	<0.0001	0.79			
	Ontario	13.126	<0.0001	0.69			
	Ontario/Nova Scotia	15.395	<0.0001	0.81			
	Strober	11.026	0.001	0.58			
	Discrete	14.052	<0.0001	0.74			
	New York	15.754	<0.0001	0.83			
	Ireland	18.916	<0.0001	1			
CVLT-II	Manitoba	2.119	0.149	0.54			
	Ontario	3.914	0.051	1			
	Discrete	2.329	0.130	0.60			
	New York	2.685	0.104	0.68			
	Ireland	3.302	0.072	0.84			
BVMT-R	Manitoba	1.246	0.267	0.77			
	Ontario	1.608	0.208	1			
	Discrete	1.449	0.231	0.90			
	New York	0.984	0.324	0.61			
	Ireland	0.977	0.325	0.61			

*SDMT, Symbol Digit Modalities Test; CVLT-II, California Verbal Learning Test; BVMTR, Brief Visual Memory Test-Revised*;

a *For these analyses disability was divided into two groups instead of three: “mild” (EDSS 0–2.5) and “moderate” (EDSS 3.0–4.5), as only 1 participant would have fallen into the category of “severe” (EDSS of 4.5 and above) used in Manitoba. Employment status not available for Nova Scotia sample. Bold indicates statistical significance*.

Similar to the findings for disability, the various norms differed in their ability to discriminate between employed and unemployed participants with MS. For the SDMT, the Manitoba norms best discriminated between employed and unemployed participants. For the CVLT-II verbal learning, the Ontario norms were the best discriminator, followed closely by the Manitoba norms which were similar with a RE of 0.95. For the BVMT-R, the discrete norms from the manual discriminated best between employed and unemployed participants. Considering only the Manitoba norms, the BVMT-R discriminated better than the SDMT, followed by the CVLT-II. This pattern was consistent for the Ontario, Ireland, and discrete norms from the manual, but not for the New York norms where the BVMT-R had the poorest discriminating ability.

In the sample of 104 MS participants from Nova Scotia, for the SDMT, the Ireland norms were best able to discriminate between the two (i.e., mild vs. moderate) disability groups (Table 5). The New York and Ontario/Nova Scotia norms had the next highest RE at 0.83 and 0.81, respectively. Regardless of the norms used, the CVLT-II verbal learning and BVMT-R total recall were unable to discriminate between mild vs. moderate disability groups. The Nova Scotia replication sample did not collect data regarding employment.

## Discussion

In this cross-sectional study, we applied a set of previously developed regression-based norms from Ontario, Canada for tests comprising the BICAMS, to an independently collected healthy sample from Manitoba, Canada to assess their generalizability. We also replicated our findings in a second, smaller, normative sample from Nova Scotia, Canada. In healthy controls, the rates of impairment differed from standard population expectations, sometimes being higher than expected and sometimes being lower. The application of regression-based norms developed in other non-Canadian English-speaking populations also produced variable impairment rates that differed from expectations, as did the discrete norms from the test manuals. All of the norms differed in their ability to discriminate between MS and healthy populations from Manitoba, and between Manitobans with MS who had differing levels of disability or employment status. The local Manitoba norms generally had better discriminating ability in the Manitoba sample than other norms, but the CVLT-II and BVMT-R were still poor at discriminating between healthy participants and participants with MS. A prior report in a Belgian sample also found that the CVLT-II did not discriminate between persons with and without MS (33). Prior studies examining the sensitivity of neuropsychological tests suggest that the SDMT discriminates best between people with and without MS (34), and the SDMT is commonly found to be the test most associated with other clinically relevant factors (3). This high sensitivity of the SDMT to cognitive impairment in MS has been attributed to its assessment of commonly affected cognitive abilities including processing speed and working memory, as well as its requirements for efficient visual scanning and oculomotor functioning (27). Overall, our findings indicate that using regional norms to interpret all BICAMS tasks is likely to be most informative.

Spearman correlations between the different norms all exceeded 0.75 and most correlations exceeded 0.90. However, concordance coefficients were lower, indicating that while the norms rank ordered participants similarly, the absolute z-scores differed. Notably, in the Manitoba and Nova Scotia samples, concordance was lowest between the norms from Ireland and the other English language norms, which were developed in regions of Canada or the United States; potentially reflecting greater cultural differences between Ireland and North America than among North American regions for this verbal memory test. A prior study found that nationality influences performance on all three BICAMS tests, even after adjusting for age and years of education (35). That study highlighted the importance of considering both the language and culture of the individual being tested and called for additional studies across countries with common languages to address the potential influences of cultural factors. An approach by which BICAMS can be validated in other languages has been recommended (10) and a systematic review in 2018 reported on the performance of BICAMS as translated from English into 11 languages, following which performance was assessed (28). However, within countries, including Canada, where inhabitants may use one or more languages and/or are members of different cultural groups, there may be a need for particular effort to ensure appropriate norms are applied.

In principle, clinicians, and researchers may choose to use discrete norms that are commercially available for the cognitive tests they employ, locally validated norms, or regression-based norms from other populations. For example, regression-based norms derived from a Canadian sample have been employed in Sweden, albeit modified to exclude educational level (36). A large multi-center trial of exercise and cognitive rehabilitation will be applying Dutch norms at the Denmark site (37). Notably, even when we employed only norms developed in other regions of Canada, our local norms, and discrete norms from the manuals for each test that are used in clinical practice, we observed meaningful variations in impairment classification rates and in the ability to discriminate between and within groups. This reflects the differences in the absolute z-scores, as demonstrated by the lower concordance coefficients than correlation coefficients. These differences may reflect differences in the healthy populations enrolled, as well as differences in the approaches used to develop the norms. For example, Walker *et al*. used raw test scores in their regression models and did not incorporate a non-linear term for age (11), while Berrigan et al. used scaled scores and incorporated a non-linear term for age that reflected non-linear findings reported in large samples (22). Our findings suggest that methodological issues such as these constitute an important component of the wide variation in the frequency of cognitive impairment reported in the MS literature [reviewed in Chiaravalloti and DeLuca (38)]. Differences in the ability to discriminate between healthy and MS groups, and between groups of persons with MS at differing levels of neurologic disability and employment status, also highlight how the use of different norms affects the identification of factors influencing cognitive outcomes.

Within the Manitoba healthy sample, the contribution of demographic characteristics to cognitive performance also varied across the three cognitive tests evaluated, with the variance explained ranging from 15 to 21%, consistent with prior reports (26). The poorer performance seen on the SDMT and BVMT-R with increasing age is consistent with prior reports in healthy populations (39, 40). Sex was associated with performance of the CVLT-II, but not the SDMT or BVMT-R. One prior report suggested that the association of sex SDMT performance is only seen for the written version of this test, with women having better scores than men, whereas this is not the case of for the oral version used here and recommended for persons with MS (39). Education was not associated with cognitive performance, but most of our healthy sample was well-educated. Race predominantly contributed to performance on the SDMT in our sample although the association between race, ethnicity and performance of cognitive tests is well-recognized (40).

Raw scores on cognitive tests have been demonstrated to have higher sensitivity than demographically-corrected scores for discriminating between persons with and without cognitive impairment, but demographically-corrected scores have higher specificity (41). Several options exist for demographically correcting scores. Discrete norms are easy to develop but require continuous variables such as age to be categorized. This creates somewhat arbitrary and discontinuous changes in expected performance for individuals at the boundaries of those categories and relatively large sample sizes are required to develop precise norms with smaller categories that address this issue (15). Regression-based norms have become popular because they do not categorize continuous variables, and the improved efficiency of estimation allows for the use of substantially smaller sample sizes while providing more precise estimates. For the BICAMS, the international validation standards recommend that the minimum sample size is 65 healthy volunteers, provided that they are group matched on demographics to an MS sample (10). Samples of ≥150 persons or more are encouraged for generalizability. We used linear regression models to develop our norms as is common in the literature. This approach is affected by whether model assumptions are met, and model assumptions were met in this study. Nonetheless, skewness may interfere with norm accuracy (42), and outliers may exert a substantial influence on the norms that are developed, particularly in smaller samples. Linear regression examines the relationship between the conditional mean of the dependent variable to the independent variables of interest, and assumes that this adequately represents relationships across the entire distribution of the dependent variable. Moreover, traditional linear regression does not account for the fact that cognitive tests typically have a limited range of scores and therefore, we employed a truncated regression model to account for this issue.

Limitations of this study should be recognized. To ensure comparability with existing Canadian-Ontario regression-based norms, we did not include participants over age 65 years. However, after restricting our analyses to persons who were aged ≤65 years, we had 96 participants for developing local norms in Manitoba. While this exceeds the minimum 65 persons recommended in the BICAMS international standards for validation (10), it is slightly <100 recommended based on simulation studies (15). Like the healthy samples used to develop regression-based norms for BICAMS that we evaluated here (Supplementary Table e1), our healthy sample predominantly included women (*n* = 32 men). Most of our study population were white, thus further work is needed to develop norms that account for the racial/ethnic diversity in Canada and elsewhere. This is particularly important as recognition grows of the burden of MS in populations traditionally considered to be at a lower risk of MS such as indigenous Canadians and African Americans (43, 44). We did not capture acculturation which may also influence performance of norms (45). On average, the healthy control sample in Manitoba was younger than the MS sample, and more highly educated; differences in sex distribution were more modest as indicated by the standardized difference of <0.20. Norms should be applied cautiously in populations with different characteristics than those in whom they were developed due to limitations in generalizability, as illustrated by our findings. However, while the samples differed on average, the age and years of education distributions overlapped.

Regression-based norms have advantages over discrete norms. However, our findings emphasize the value of local norms when interpreting the findings of cognitive tests (46) and demonstrate the need to consider and assess the performance of regression-based norms developed in other populations when applying them to local populations, even when they are from the same country. This is important to avoid misclassifying individuals as to whether they are cognitively impaired or unimpaired. Our findings also strongly suggest that the development of regression-based norms should involve larger, more diverse samples to ensure broad generalizability. Specifically, greater representation is needed of men, individuals over age 65 years, and of varying racial, ethnic, and social backgrounds.

## Data Availability Statement

The datasets presented in this article are not readily available because some participants did not agree to data sharing; some data may be accessible to qualified investigators with the appropriate ethical approvals and data use agreements. Requests to access the datasets should be directed to Ruth Ann Marrie, rmarrie@hsc.mb.ca.

## Ethics Statement

The studies involving human participants were reviewed and approved by the University of Manitoba Health Research Ethics Board and the Nova Scotia Health Authority Research Ethics Board. The patients/participants provided their written informed consent to participate in this study.

## Author Contributions

RM, JF, and RP conceived of the idea. RM, RP, CF, JK JB, LG, EM, JM, CB, and JF obtained study funding. RM and CW conducted the statistical analyses and drafted the manuscript. RM, RP, CW, CF, JK, JB, LG, EM, JM, CB, and JF revised the manuscript and approved of the final version. All authors contributed to the article and approved the submitted version.

## Conflict of Interest

RM receives research funding from: CIHR, Research Manitoba, Multiple Sclerosis Society of Canada, Multiple Sclerosis Scientific Foundation, Crohn's and Colitis Canada, National Multiple Sclerosis Society, CMSC, the Arthritis Society, and US Department of Defense. CW was supported by a Canadian Institutes of Health Research Frederick Banting and Charles Best Canada Graduate Scholarship Doctoral Award. RP receives research funding from the Workers Compensation Board of Manitoba. CF receives research funding from the Brain Canada Foundation, MS Society of Canada, Natural Sciences and Engineering Research Council of Canada, and Health Sciences Center Foundation. JK receives research funding from the MS Society of Canada, Crohn's and Colitis Canada, University of Manitoba, and Health Sciences Center Foundation. JB receives research funding from CIHR, Brain, and Behavior Research Foundation and the MS Society of Canada. LG receives research funding from: CIHR, the MS Society of Canada, Crohns and Colitis Canada, and the Health Sciences Center Foundation. EM received fellowship funding from NSERC and Alberta Innovates-Health Solutions and receives research funding from the MS Society of Canada. JM has conducted trials for Biogen Idec and Roche, and receives research funding from the MS Society of Canada. CB is supported in part by the Bingham Chair in Gastroenterology. He serves on Advisory Boards for Abbvie Canada, Janssen Canada, Takeda Canada, Pfizer Canada. He is a Consultant for Mylan Pharmaceuticals. He is receiving educational grants from Abbvie Canada, Pfizer Canada, Shire Canada, Takeda Canada, Janssen Canada. Speaker's panel for Abbvie Canada, Janssen Canada, Takeda Canada, and Medtronic Canada. He received research funding from Abbvie Canada. JF receives research funding from: CIHR, Multiple Sclerosis Society of Canada, Crohn's and Colitis Canada, the Nova Scotia Health Authority Research Fund; consultation and distribution royalties from MAPI Research Trust.

## References

[1] Goverover Y Strober L Chiaravalloti N DeLuca J. Factors that moderate activity limitation and participation restriction in people with multiple sclerosis. Am J Occup Ther. (2015) 69:1–9. 10.5014/ajot.2015.014332 26122682

[2] Rao SM Leo GJ Ellington L Nauertz T Bernardin L Unverzagt F. Cognitive dysfunction in multiple sclerosis II: impact on employment and social functioning. Neurology. (1991) 41:692–6. 10.1212/WNL.41.5.692 1823781

[3] Moccia M Lanzillo R Palladino R Chang KCM Costabile T Russo C. Cognitive impairment at diagnosis predicts 10-year multiple sclerosis progression. Mult Sclerosis J. (2016) 22:659–67. 10.1177/1352458515599075 26362896

[4] Langdon DW Amato MP Boringa J Brochet B Foley F Fredrikson S. Recommendations for a brief international cognitive assessment for multiple sclerosis (BICAMS). Mult Scler. (2012) 18:891–8. 10.1177/1352458511431076 22190573 PMC3546642

[5] Boringa JB Lazeron RH Reuling IE Adèr HJ Pfennings LE Lindeboom J. The brief repeatable battery of neuropsychological tests: normative values allow application in multiple sclerosis clinical practice. Mult Scler. (2001) 7:263–7. 10.1177/135245850100700409 11548987

[6] Benedict RHB Fischer JS Archibald CJ Arnett PA Beatty WW Bobholz J Minimal neuropsychological assessment of MS patients: a consensus approach. Clin Neuropsychol. (2002) 16:381–97. 10.1076/clin.16.3.381.13859 12607150

[7] Chevalier TM Stewart G Nelson M McInerney RJ Brodie N Impaired or not impaired, that is the question: navigating the challenges associated with using canadian normative data in a comprehensive test battery that contains American tests. Arch Clin Neuropsychol. (2016) 31:446–55. 10.1093/arclin/acw031 27246955 PMC4954611

[8] Trahan LH Stuebing KK Fletcher JM Hiscock M. The flynn effect: a meta-analysis. Psychol Bull. (2014) 140:1332–60. 10.1037/a0037173 24979188 PMC4152423

[9] Au R Seshadri S Wolf PA Elias M Elias P Sullivan L. New norms for a new generation: cognitive performance in the framingham offspring cohort. Exp Aging Res. (2004) 30:333–58. 10.1080/03610730490484380 15371099

[10] Benedict RHB Amato MP Boringa J Brochet B Foley F Fredrikson S. Brief international cognitive assessment for MS (BICAMS): international standards for validation. BMC Neurol. (2012) 12:55. 10.1186/1471-2377-12-55 22799620 PMC3607849

[11] Walker LAS Marino D Berard JA Feinstein A Morrow SA Cousineau D. Canadian normative data for minimal assessment of cognitive function in multiple sclerosis. Can J Neurol Sci. (2017) 44:547–55. 10.1017/cjn.2017.199 28683843

[12] Marrie RA Graff LA Walker JR Fisk JD Patten SB Hitchon CA. A prospective study of the effects of psychiatric comorbidity in immune-mediated inflammatory disease: rationale, protocol and participation. JMIR Res Protoc. (2018) 7:e15. 10.2196/resprot.8794 29343461 PMC5792704

[13] Marrie RA Patel R Figley CR Kornelsen J Bolton JM Graff L. Diabetes and anxiety adversely affect cognition in multiple sclerosis. Mult Scler Relat Disord. (2019) 27:164–70. 10.1016/j.msard.2018.10.018 30384203

[14] Mazziotta JC Woods R Iacoboni M Sicotte N Yaden K Tran M. The myth of the normal, average human brain-The ICBM experience: (1) subject screening and eligibility. NeuroImage. (2009) 44:914–22. 10.1016/j.neuroimage.2008.07.062 18775497 PMC2651672

[15] Oosterhuis HE van der Ark LA Sijtsma K. Sample size requirements for traditional and regression-based norms. Assessment. (2016) 23:191–202. 10.1177/1073191115580638 25940350

[16] Grant BF Hasin DS Chou S Stinson FS Dawson DA. Nicotine dependence and psychiatric disorders in the United States: results from the national epidemiologic survey on alcohol and related conditions. Arch Gen Psychiatry. (2004) 61:1107–15. 10.1001/archpsyc.61.11.1107 15520358

[17] van den Berg E Kloppenborg RP Kessels RP Kappelle LJ Biessels GJ. Type 2 diabetes mellitus, hypertension, dyslipidemia and obesity: a systematic comparison of their impact on cognition. Biochim Biophys Acta. (2009) 1792:470–81. 10.1016/j.bbadis.2008.09.004 18848880

[18] Genova HM Sumowski JF Chiaravalloti N Voelbel GT Deluca J. Cognition in multiple sclerosis: a review of neuropsychological and fMRI research. Front Biosci. (2009) 14:1730–44. 10.2741/3336 19273158

[19] Smith A Symbol Digit Modalities Test. 9th ed Torrance, CA: Western Psychological Services (2002)

[20] Delis DC Kramer JH Kaplan E Ober BA California Verbal Learning Test Second Edition Adult Version Manual. San Antonio, TX: The Psychological Corporation (2000).

[21] Benedict RHB Hopkins BJ Verbal Learning Test-Revised/Brief Visuospatial Memory Test-Revised Professional Manual Supplement. Odessa, FL: Psychological Assessment Resources (2001).

[22] Berrigan LI Fisk JD Walker LA Wojtowicz M Rees LM Freedman MS. Reliability of regression-based normative data for the oral symbol digit modalities test: an evaluation of demographic influences, construct validity, and impairment classification rates in multiple sclerosis samples. Clin Neuropsychol. (2014) 28:281–99. 10.1080/13854046.2013.871337 24438521

[23] Snitz BE Unverzagt FW Chang CCH Bilt JV Gao S Saxton J. Effects of age, gender, education and race on two tests of language ability in community-based older adults. Int Psychogeriatr. (2009) 21:1051–62. 10.1017/S1041610209990214 19586563 PMC2783556

[24] Pedraza O Graff-Radford NR Smith GE Ivnik RJ Willis FB Petersen RC. Differential item functioning of the boston naming test in cognitively normal African American and Caucasian older adults. J Int Neuropsychol Soc. (2009) 15:758–68. 10.1017/S1355617709990361 19570311 PMC2835360

[25] Long JS. Regression Models for Categorical and Limited Dependent Variables. Thousand Oaks, CA: Sage Publications (1997).

[26] Parmenter BA Testa SM Schretlen DJ Weinstock-Guttman B Benedict RHB. The utility of regression-based norms in interpreting the minimal assessment of cognitive function in multiple sclerosis (MACFIMS). J Int Neuropsychol Soc. (2010) 16:6–16. 10.1017/S1355617709990750 19796441

[27] Strober LB Bruce JM Arnett PA Alschuler KN Lebkuecher A Di Benedetto M A new look at an old test: normative data of the symbol digit modalities test -Oral version. Mult Scler Relat Disord. (2020) 43:102154 10.1016/j.msard.2020.102154 32450507

[28] Corfield F Langdon D. A systematic review and meta-analysis of the brief cognitive assessment for multiple sclerosis (BICAMS). Neurol Ther. (2018) 7:287–306. 10.1007/s40120-018-0102-3 29923070 PMC6283796

[29] Swinscow T 11 Correlation and regression. Statistics at Square One. London: BMJ Publishing Group (1997).

[30] Lin LI. A concordance correlation coefficient to evaluate reproducibility. Biometrics. (1989) 45:255–68. 10.2307/2532051 2720055

[31] Ruano L Portaccio E Goretti B Niccolai C Severo M Patti F. Age and disability drive cognitive impairment in multiple sclerosis across disease subtypes. Mult Scler. (2017) 23:1258–67. 10.1177/1352458516674367 27738090

[32] Zhang W Bansback N Boonen A Young A Singh A Anis AH. Validity of the work productivity and activity impairment questionnaire–general health version in patients with rheumatoid arthritis. Arthritis Res Ther. (2010) 12:R177. 10.1186/ar3141 20860837 PMC2991008

[33] Costers L Gielen J Eelen PL Schependom JV Laton J Remoortel AV. Does including the full CVLT-II and BVMT-R improve BICAMS? Evidence from a Belgian (Dutch) validation study. Mult Scler Relat Disord. (2017) 18:33–40. 10.1016/j.msard.2017.08.018 29141818

[34] Strober L Englert J Munschauer F Weinstock-Guttman B Rao S Benedict R. Sensitivity of conventional memory tests in multiple sclerosis: comparing the rao brief repeatable neuropsychological battery and the minimal assessment of cognitive function in MS. Mult Sclerosis J. (2009) 15:1077–84. 10.1177/1352458509106615 19556311

[35] Smerbeck A Benedict RHB Eshaghi A Vanotti S Spedo C Blahova Dusankova J. Influence of nationality on the brief international cognitive assessment for multiple sclerosis (BICAMS). Clin Neuropsychol. (2018) 32:54–62. 10.1080/13854046.2017.1354071 28721748

[36] McKay KA Manouchehrinia A Berrigan L Fisk JD Olsson T Hillert J. Long-term cognitive outcomes in patients with pediatric-onset vs adult-onset multiple sclerosis. JAMA Neurol. (2019) 76:1028–34. 10.1001/jamaneurol.2019.1546 31206130 PMC6580443

[37] Feinstein A Amato MP Brichetto G Chataway J Chiaravalloti N Dalgas U. Study protocol: improving cognition in people with progressive multiple sclerosis: a multi-arm, randomized, blinded, sham-controlled trial of cognitive rehabilitation and aerobic exercise (COGEx). BMC Neurol. (2020) 20:204. 10.1186/s12883-020-01772-7 32443981 PMC7245035

[38] Chiaravalloti ND DeLuca J. Cognitive impairment in multiple sclerosis. Lancet Neurol. (2008) 7:1139–51. 10.1016/S1474-4422(08)70259-X 19007738

[39] Fellows RP Schmitter-Edgecombe M. Symbol digit modalities test: regression-based normative data and clinical utility. Arch Clin Neuropsychol. (2019) 35:105–15. 10.1093/arclin/acz020 31329813

[40] Norman MA Moore DJ Taylor M Franklin D Jr Cysique L. Demographically corrected norms for African Americans and caucasians on the hopkins verbal learning test-revised, brief visuospatial memory test-revised, stroop color and word test, and wisconsin card sorting test 64-card version. J Clin Exp Neuropsychol. (2011) 33:793–804. 10.1080/13803395.2011.559157 21547817 PMC3154384

[41] O'Connell ME Tuokko H. Age corrections and dementia classification accuracy. Arch Clin Neuropsychol. (2010) 25:126–38. 10.1093/arclin/acp111 20118110

[42] O'Connell ME Tuokko H Kadlec H. Demographic corrections appear to compromise classification accuracy for severely skewed cognitive tests. J Clin Exp Neuropsychol. (2011) 33:422–31. 10.1080/13803395.2010.532114 21154077

[43] Svenson LW Warren S Warren KG Metz LM Patten SB Schopflocher DP. Prevalence of multiple sclerosis in first nations people of Alberta. Can J Neurol Sci. (2007) 34:175–80. 10.1017/S0317167100006004 17598594

[44] Langer-Gould A Brara SM Beaber BE Zhang JL. Incidence of multiple sclerosis in multiple racial and ethnic groups. Neurology. (2013) 80:1734–9. 10.1212/WNL.0b013e3182918cc2 23650231

[45] Kennepohl S Shore D Nabors N Hanks R. African American acculturation and neuropsychological test performance following traumatic brain injury. J Int Neuropsychol Soc. (2004) 10:566–77. 10.1017/S1355617704104128 15327735

[46] Anderson SJ On the importance of collecting local neuropsychological normative data. South African J Psychol. (2001) 31:29–34. 10.1177/008124630103100304

