# Supplementary material for: Performance of Regression-Based Norms for Cognitive Functioning of Persons With Multiple Sclerosis in an Independent Sample
Source: Front Neurol. 2021 Jan 14;11:621010. doi: 10.3389/fneur.2020.621010 (PMC7840703; doi:10.3389/fneur.2020.621010)
Supplement: Supplementary file 1 [file Table_1.DOCX]

Supplementary Material

Table e1. Characteristics of the normative samples and approaches used to develop the norms tested in the present study

| **Norm** | **Cognitive**  **Test** | **Year** | **Region** | **Sample Size** | **Age Range**  **(Years)** | **Scores Used** | **Non-linear term for age** |
| --- | --- | --- | --- | --- | --- | --- | --- |
| Berrigan | SDMT | 2013 | Ottawa, Ontario &  Nova Scotia, Canada | 94 (Gender NR) | 20-65 | Raw scores converted to scaled scores (M = 10, SD = 3) using the cumulative percent of frequency distribution | Yes*  *Age was centred |
| Walker | SDMT  CVLT free  CVLT LD  BVMT-R  BVMT-R DR | 2017 | Ontario (3 centres), Canada | 328 (244 F)  108 (84 F)  57 (40 F)  180 (143 F)  129 (99 F) | 18-65  20-65  22-65  20-65  20-65 | Raw scores | No |
| Parmenter | SDMT  CVLT free  CVLT LD  BVMT-R | 2010 | Buffalo, New York, USA | 100 (79 F) | Range NR  Mean (SD): 44.79 (9.43) | Raw scores converted to scaled scores (M = 10, SD = 3) using the cumulative percent of frequency distribution | Yes |
| O’Connell | SDMT  CVLT  BVMT-R | 2015 | Dublin, Ireland | 66 (45 F) | Range NR  Mean (SD):  42.7 (12.8) | Raw scores converted to scaled scores (M = 10, SD = 3) using the cumulative percent of frequency distribution | Yes |
| *Discrete Norms* |  |  |  |  |  |  |  |
| Strober | SDMT | 2020 | New Jersey, Pennsylvania, Missouri, Washington, USA | 675 (354 F) | 18-74 | N/A | N/A |
| Smith | SDMT | 1982 | New Jersey & Michigan, USA | 1307 (gender NR but reportedly evenly distributed) | 18-78 | N/A | N/A |
| Delis | CVLT | 2000 | 47 states, USA | 1087 | 16-89 | N/A | N/A |
| Benedict | BVMT-R | 2007 | Northeastern USA | 588 (378 F) | 18-79 | N/A | N/A |

BVMT-R = Brief Visuospatial Memory Test-Revised, CVLT = California Verbal Learning Test-II, SDMT = Symbol Digit Modalities Test, NR = Not reported

Table e2. Spearman two-tailed correlation coefficients and concordance coefficients for the association between different norms in Nova Scotia.

|  | **Healthy Controls (n = 39)** | | **Multiple Sclerosis (n = 104)** | |
| --- | --- | --- | --- | --- |
|  | **Correlation**  **coefficient**  **(95%CI)** | **Concordance coefficient**  **(95%CI)** | **Correlation**  **coefficient**  **(95%CI)** | **Concordance coefficient**  **(95%CI)** |
| ***SDMT*** |  |  |  |  |
| SDMT_NY_ – SDMT_IRE_ | 0.89 (0.80, 0.94) | 0.42 (0.29, 0.54) | 0.95 (0.93, 0.97) | 0.67 (0.60, 0.74) |
| SDMT_NY_ – SDMT_ONT_ | 0.96 (0.92, 0.98) | 0.87 (0.80, 0.92) | 0.98 (0.98, 0.99) | 0.91 (0.88, 0.93) |
| SDMT_NY_ – SDMT _ONT/NS_ | 0.96 (0.93, 0.98) | 0.92 (0.86, 0.96) | 0.99 (0.98, 0.99) | 0.94 (0.92, 0.96) |
| SDMT_IRE_ – SDMT_ONT_ | 0.89 (0.80, 0.94) | 0.53 (0.38, 0.65) | 0.96 (0.94, 0.97) | 0.81 (0.75, 0.85) |
| SDMT_IRE_ – SDMT _ONT/NS_ | 0.88 (0.77, 0.93) | 0.51 (0.36, 0.63) | 0.95 (0.93, 0.96) | 0.76 (0.69, 0.82) |
| SDMT_ONT_ – SDMT _ONT/NS_ | 0.98 (0.96, 0.99) | 0.97 (0.94, 0.98) | 0.98 (0.98, 0.99) | 0.97 (0.96, 0.98) |
| SDMT_NY_ - SDMT_DISCRETE_ | 0.94 (0.89, 0.97) | 0.80 (0.70, 0.88) | 0.97 (0.96, 0.98) | 0.86 (0.81, 0.89) |
| SDMT_NY_ – SDMT_STROBER_ | 0.95 (0.91, 0.97) | 0.90 (0.82, 0.94) | 0.96 (0.94, 0.97) | 0.94 (0.92, 0.96) |
| SDMT_IRE_ - SDMT_DISCRETE_ | 0.90 (0.81, 0.95) | 0.58 (0.43, 0.69) | 0.96 (0.94, 0.97) | 0.83 (0.78, 0.87) |
| SDMT_IRE_ – SDMT_STROBER_ | 0.84 (0.72, 0.92) | 0.48 (0.32, 0.61) | 0.91 (0.86, 0.94) | 0.71 (0.63, 0.78) |
| SDMT_ONT_ – SDMT_DISCRETE_ | 0.96 (0.93, 0.98) | 0.95 (0.90, 0.97) | 0.98 (0.98, 0.99) | 0.97 (0.96, 0.98) |
| SDMT_ONT_ – SDMT_STROBER_ | 0.94 (0.90, 0.97) | 0.85 (0.76, 0.91) | 0.96 (0.94, 0.97) | 0.94 (0.91, 0.96) |
| SDMT_NS_ – SDMT_DISCRETE_ | 0.96 (0.93, 0.98) | 0.90 (0.83, 0.94) | 0.97 (0.96, 0.98) | 0.94 (0.92, 0.96) |
| SDMT_NS_ – SDMT_STROBER_ | 0.95 (0.91, 0.98) | 0.88 (0.80, 0.94) | 0.96 (0.94, 0.97) | 0.94 (0.92, 0.96) |
| SDMT_DISCRETE_ – SDMT_STROBER_ | 0.94 (0.89, 0.97) | 0.83 (0.74, 0.90) | 0.96 (0.94, 0.97) | 0.91 (0.88, 0.94) |
| SDMT_MAN_ – SDMT_IRE_ | 0.83 (0.70, 0.91) | 0.39 (0.25, 0.52) | 0.93 (0.89, 0.95) | 0.71 (0.63, 0.77) |
| SDMT_MAN_ – SDMT_ONT_ | 0.96 (0.93, 0.98) | 0.88 (0.80, 0.92) | 0.98 (0.97, 0.99) | 0.96 (0.94, 0.97) |
| SDMT_MAN_ – SDMT_NY_ | 0.91 (0.84, 0.95) | 0.88 (0.79, 0.94) | 0.95 (0.93, 0.97) | 0.93 (0.90, 0.95) |
| SDMT_MAN_ – SDMT_DISCRETE_ | 0.92 (0.85, 0.96) | 0.76 (0.63, 0.85) | 0.97 (0.96, 0.98) | 0.91 (0.87, 0.94) |
| SDMT_MAN_ – SDMT_STROBER_ | 0.92 (0.85, 0.96) | 0.82 (0.69, 0.90) | 0.96 (0.93, 0.97) | 0.94 (0.91, 0.96) |
| SDMT_MAN_ – SDMT_NS_ | 0.94 (0.89, 0.97) | 0.92 (0.85, 0.95) | 0.96 (0.93, 0.97) | 0.96 (0.94, 0.97) |
| ***CVLT*** |  |  |  |  |
| CVLT_NY_ - CVLT_IRE_ | 0.68 (0.46, 0.82) | 0.20 (0.10, 0.30) | 0.78 (0.70, 0.85) | 0.35 (0.27, 0.43) |
| CVLT_NY_ – CVLT_ONT_ | 0.90 (0.82, 0.95) | 0.51 (0.37, 0.62) | 0.91 (0.87, 0.94) | 0.65 (0.57, 0.72) |
| CVLT_IRE_ – CVLT_ONT_ | 0.87 (0.76, 0.93) | 0.56 (0.41, 0.68) | 0.95 (0.93, 0.97) | 0.76 (0.70, 0.82) |
| CVLT_NY_ – CVLT_DISCRETE_ | 0.85 (0.73, 0.92) | 0.28 (0.17, 0.38) | 0.89 (0.84, 0.92) | 0.38 (0.30, 0.45) |
| CVLT_IRE_ – CVLT_DISCRETE_ | 0.87 (0.76, 0.93) | 0.79 (0.66, 0.88) | 0.89 (0.84, 0.92) | 0.87 (0.82, 0.90) |
| CVLT_ONT_ – CVLT_DISCRETE_ | 0.90 (0.82, 0.95) | 0.69 (0.55, 0.79) | 0.93 (0.90, 0.95) | 0.76 (0.70, 0.82) |
| CVLT_MAN_ – CVLT_NY_ | 0.97 (0.95, 0.99) | 0.50 (0.37, 0.61) | 0.97 (0.95, 0.98) | 0.56 (0.48, 0.63) |
| CVLT_MAN_ – CVLT_IRE_ | 0.72 (0.52, 0.84) | 0.48 (0.29, 0.63) | 0.82 (0.74, 0.87) | 0.73 (0.64, 0.80) |
| CVLT_MAN_ – CVLT_ONT_ | 0.94 (0.89, 0.97) | 0.93 (0.87, 0.96) | 0.94 (0.91, 0.96) | 0.91 (0.88, 0.94) |
| CVLT_MAN_ – CVLT_DISCRETE_ | 0.87 (0.76, 0.93) | 0.70 (0.56, 0.81) | 0.91 (0.87, 0.94) | 0.84 (0.78, 0.88) |
| ***BVMTR*** |  |  |  |  |
| BVMTR_NY_ - BVMTR_IRE_ | 0.91 (0.84, 0.95) | 0.48 (0.34, 0.59) | 0.94 (0.91, 0.96) | 0.40 (0.33, 0.47) |
| BVMTR_NY_ – BVMTR_ONT_ | 0.97 (0.94, 0.98) | 0.92 (0.87, 0.96) | 0.95 (0.93, 0.97) | 0.88 (0.83, 0.91) |
| BVMTR_IRE_ – BVMTR_ONT_ | 0.96 (0.92, 0.98) | 0.58 (0.45, 0.68) | 0.98 (0.98, 0.99) | 0.56 (0.48, 0.62) |
| BVMTR_NY_ – BVMTR_DISCRETE_ | 0.97 (0.94, 0.98) | 0.87 (0.79, 0.92) | 0.93 (0.90, 0.95) | 0.81 (0.75, 0.85) |
| BVMTR_IRE_ – BVMTR_DISCRETE_ | 0.94 (0.90, 0.97) | 0.68 (0.56, 0.77) | 0.98 (0.97, 0.99) | 0.64 (0.58, 0.71) |
| BVMTR_ONT_ – BVMTR_DISCRETE_ | 0.99 (0.99, 1.00) | 0.97 (0.96, 0.98) | 0.99 (0.99, 0.99) | 0.97 (0.96, 0.98) |
| BVMTR_MAN_ – BVMTR_NY_ | 0.96 (0.93, 0.98) | 0.86 (0.77, 0.91) | 0.93 (0.90, 0.95) | 0.81 (0.75, 0.86) |
| BVMTR_MAN_ – BVMTR_IRE_ | 0.93 (0.86, 0.96) | 0.62 (0.48, 0.73) | 0.96 (0.93, 0.97) | 0.56 (0.48, 0.63) |
| BVMTR_MAN_ – BVMTR_ONT_ | 0.98 (0.96, 0.99) | 0.95 (0.91, 0.97) | 0.98 (0.96, 0.98) | 0.95 (0.93, 0.97) |
| BVMTR_MAN_ – BVMTR_DISCRETE_ | 0.99 (0.99, 1.00) | 0.97 (0.95, 0.98) | 0.99 (0.98, 0.99) | 0.96 (0.95, 0.97) |

Figure e1. Receiver Operating Characteristic Curves for Cognitive Test Norms Comparing Persons with and without Multiple Sclerosis in Nova Scotia: (A) SDMT (B) CVLT-II (C) BVMT-R

A.

B.

C.
